# Physiological and molecular effects of interleukin-18 administration on the mouse kidney

**DOI:** 10.1186/s12967-018-1426-6

**Published:** 2018-03-07

**Authors:** Kyosuke Yamanishi, Keiichiro Mukai, Takuya Hashimoto, Kaoru Ikubo, Keiji Nakasho, Yosif El-Darawish, Wen Li, Daisuke Okuzaki, Yuko Watanabe, Tetsu Hayakawa, Hiroshi Nojima, Hiromichi Yamanishi, Haruki Okamura, Hisato Matsunaga

**Affiliations:** 1 Department of Neuropsychiatry, Hyogo College of Medicine, 1-1 Mukogawa, Nishinomiya, Hyogo 663-8501 Japan; 2 Department of Pathology, Hyogo College of Medicine, 1-1 Mukogawa, Nishinomiya, Hyogo 663-8501 Japan; 3 Laboratory of Tumor Immunology and Cell Therapy, Hyogo College of Medicine, 1-1 Mukogawa, Nishinomiya, Hyogo 663-8501 Japan; 4 DNA-Chip Development Center for Infectious Diseases, Research Institute for Microbial Diseases, Osaka University, 3-1 Yamadaoka, Suita, 565-0871 Japan; 5 Department of Molecular Genetics, Research Institute for Microbial Diseases, Osaka University, 3-1 Yamadaoka, Suita, 565-0871 Japan; 6 Hirakata General Hospital for Developmental Disorders, 2-1-1 Tsudahigashi, Hirakata, Osaka 573-0122 Japan

**Keywords:** Interleukin-18, Interleukin-18 knockout, Kidney, Clinical translation, Kidney injury, Microarray, Quantitative reverse transcription polymerase chain reaction

## Abstract

**Background:**

The cytokine interleukin-18 was originally identified as an interferon-γ-inducing proinflammatory factor; however, there is increasing evidence to suggest that it has non-immunological effects on physiological functions. We previously investigated the potential pathophysiological relationship between interleukin-18 and dyslipidemia, non-alcoholic fatty liver disease, and non-alcoholic steatohepatitis, and suggested interleukin-18 as a possible novel treatment for not only these diseases but also for cancer immunotherapy. Before clinical application, the effects of interleukin-18 on the kidney need to be determined. In the current study, we examined the kidney of interleukin-18 knockout (*Il18*^−/−^) mice and the effects of interleukin-18 on the kidney following intravenous administration of recombinant interleukin-18.

**Methods:**

*Il18*^−/−^ male mice were generated on the C57Bl/6 background and littermate C57Bl/6 *Il18*^+/+^ male mice were used as controls. To assess kidney damage, serum creatinine and blood urea nitrogen levels were measured and histopathological analysis was performed. For molecular analysis, microarray and quantitative reverse transcription PCR was performed using mice 6 and 12 weeks old. To evaluate the short- and long-term effects of interleukin-18 on the kidney, recombinant interleukin-18 was administered for 2 and 12 weeks, respectively.

**Results:**

Compared with *Il18*^+/+^ mice, *Il18*^−*/*−^ mice developed kidney failure in their youth-6 weeks of age, but the condition was observed to improve as the mice aged, even though dyslipidemia, arteriosclerosis, and higher insulin resistance occurred. Analyses of potential molecular mechanisms involved in the onset of early kidney failure in *Il18*^−/−^ mice identified a number of associated genes, such as *Itgam*, *Nov*, and *Ppard*. Intravenous administration of recombinant interleukin-18 over both the short and long term showed no effects on the kidney despite significant improvement in metabolic diseases.

**Conclusions:**

Short- and long-term administration of interleukin-18 appeared to have no adverse effects on the kidney in these mice, suggesting that administration may be a safe and novel treatment for metabolic diseases and cancer.

**Electronic supplementary material:**

The online version of this article (10.1186/s12967-018-1426-6) contains supplementary material, which is available to authorized users.

## Background

The cytokine interleukin-18 (IL-18) was originally identified as an interferon-γ-inducing proinflammatory factor; however, there is increasing evidence to support its non-immunological effects on physiological functions [1–3]. IL-18 is produced as an inactive 24-kDa precursor and is processed by inflammasomes to an active 18-kDa mature form [4–7]. Previous studies have reported that mice deficient in IL-18 developed hyperphagia, obesity, and insulin resistance [8]. IL-18-knockout (*Il18*^−/−^) mice also showed dyslipidemia, non-alcoholic fatty liver disease (NAFLD), or non-alcoholic steatohepatitis (NASH) [3]. In human studies, the serum concentration of IL-18 was found to be significantly higher in patients with metabolic syndrome, type 2 diabetes mellitus, or diabetic nephropathy compared with healthy control participants [9–11].

The development of structural and functional changes in the kidney in patients with diabetes mellitus has been known for more than 20 years [12]. Diabetic nephropathy is considered the most frequent cause of end-stage renal failure in the United States [13]. A previous study found that *Il18*^−/−^ mice showed severe insulin resistance resulting in diabetes mellitus [8]; however, specific renal complications remain uncertain.

We previously reported on a novel cancer immunotherapy with IL-18 [14]. Intravenous administration of recombinant IL-18 (rIL-18) for *Il18*^−/−^ mice significantly improved dyslipidemia and prevented the onset of NASH. IL-18 may therefore be a promising factor that will contribute to novel treatment options for NAFLD or NASH mainly through correction of energy unbalances by lipids or glucose in the liver [3]. It is important that before clinical application, possible side effects of rIL-18 on the kidney are examined.

This study investigated whether *Il18*^−/−^ mice develop kidney failure as they age and whether IL-18 has beneficial or damaging effects on the kidney. We analyzed the role of IL-18 in the kidney by histopathological observation and by measuring serum concentrations of several markers in *Il18*^−/−^ mice during growth. The molecular mechanisms affected during growth were analyzed and the influence of short- and long-term administration of rIL-18 was assessed.

## Methods

### Animals

*Il18*^−/−^ male mice were generated on the C57Bl/6 background as previously described [15]. Littermate C57Bl/6 *Il18*^+/+^ male mice were used as controls. Mice were housed in groups of 3–5 in polycarbonate cages in a colony room that was maintained at a constant temperature (22 ± 1 °C) and humidity (50–60%) on a 12-h light/dark cycle (lights on at 8 a.m.) with free access to standard food (MF; Oriental Yeast Co., Ltd., Tokyo, Japan) and water. Mice were killed at 10 a.m. were used and Samples from *Il18*^+/+^ and *Il18*^−/−^ mice were taken for molecular, biochemical, and histological analyses at the same time points (n = 4–11). Additionally, five to six and three mice per group were included in the short- and long-term rIL-18 treatment groups, respectively. Details of the rIL-18 treatment are given in “Short- and long-term treatment of mice with rIL-18” section.

Animal experiments were conducted according to the “Guide for Care and Use of Laboratory Animals” published by the National Institutes of Health and approved by the Animal Care Committee of Hyogo College of Medicine (#28041 and #14-020).

### Histological analysis

Three to four mice per group were used for histopathological analysis. Mice were anesthetized with isoflurane and perfused in a transcardial manner with periodate–lysine-paraformaldehyde fixative at 10 a.m. Fixed kidneys were removed and immersed in the same fixative at 4 °C overnight. Specimens were processed for histological staining. Paraffin-embedded sections were used for hematoxylin–eosin, periodic acid–Schiff, azan, and periodic acid methenamine silver staining (all Muto Pure Chemicals Co., Ltd., Tokyo, Japan). Staining was performed according to the manufacturer’s instructions detection was achieved using the VECTASTAIN ABC Standard Kit (PK-4000; Vector Laboratories, INC., Burlingame, CA, USA) according to the manufacturer’s instructions. Paraffin-embedded sections were also stained with primary antibodies against F4/80 [product T-2008 (lot 20PO0309); dianova GmbH, Hamburg, Germany] and CD4 (553043; BD Biosciences, Tokyo, Japan). The antigen retrieval methods of F4/80 and CD4 were 4 µg/ml proteinase K (Nacalai Tesque, Inc., Kyoto, Japan) treatment at room temperature for 10 min and 0.01 M citrate buffer pH 6.0 for 10 min at 100 °C respectively referring to manufacturer’s instructions. Antibodies were diluted 1:200 and 1:125, respectively, and 5 µg/ml was used for an overnight incubation at 4 °C according to the manufacturer’s instructions, followed by incubation with 1.5 µg/ml rabbit anti-rat secondary antibody (BA-4000; Vector Laboratories, Inc.) diluted 1:1000. Stained tissues were mounted and pathological diagnosis was determined in a blind fashion by pathological specialists. Tissues were photographed using an optical microscope and CCD camera (AX-80 and DP-71; Olympus, Tokyo, Japan).

### Serum analysis

Levels of creatinine (CREA) and blood urea nitrogen (BUN) in sera were measured using enzymatic methods. Serum analysis was performed by LSI Medience Corp., Tokyo, Japan.

### Molecular analysis

The protocols for sample collection, mRNA purification, microarray, Ingenuity^®^ Pathway Analysis (IPA; Ingenuity^®^ Systems; http://www.ingenuity.com), and quantitative reverse transcription polymerase chain reaction (RT-qPCR) have been described previously [3, 16]. Mice were euthanized by decapitation at 10 a.m. and the kidneys were removed and immediately placed in liquid nitrogen and stored at − 80 °C until use.

Total RNA was purified from 12 samples using a miRNeasy mini kit (Qiagen, Hilden, Germany) according to the manufacturer’s instructions, and treated with five units of RNase free DNase I at 37 °C for 30 min to remove genomic DNA contamination. After phenol/chloroform extraction and ethanol precipitation, total RNA was dissolved in de-ionized distilled water. RNA concentrations were determined by NanoDrop-1000 spectrophotometry (NanoDrop Technologies, Wilmington, DE, USA).

For microarray analysis, expression profiling was performed using a SurePrint G3 Mouse GE 8x60K Microarray G4852A (Agilent Technologies, Inc., Santa Clara, CA, USA). Twelve microarrays (three for *Il18*^+/+^ mice and three for *Il18*^−/−^ mice at both 6 and 12 weeks of age) for one color experiment were performed as biological triplicates. Each gene expression profile was compared between *Il18*^+/+^ and *Il18*^−/−^ mice at 6 and 12 weeks of age. Total RNA (200 ng) was reverse-transcribed into double-stranded cDNA using AffinityScript multiple temperature reverse transcriptase (Agilent Technologies, Inc.) and amplified. The resulting cDNA was used for in vitro transcription by T7-polymerase and labeled with cyanine-3-labeled cytosine triphosphate (Perkin Elmer, Wellesley, MA, USA) using a Low Input Quick-Amp Labeling Kit (Agilent Technologies, Inc.). After labeled cDNA had been fragmented, each cRNA sample was hybridized on a SurePrint G3 Mouse GE 8x60K Microarray (#028005; Agilent Technologies, Inc.). After washing, slides were scanned with a microarray scanner (G2505C; Agilent Technologies, Inc.). Feature extraction software (ver. 10.5.1.1) was used to convert images into gene expression data. For microarray data analysis, raw data were imported into the Subio platform (ver. 1.18; Subio Inc., Kagoshima, Japan), and raw intensity data were normalized to the 75th‰ intensity of probes above background levels (gIsWellAbove = 1). Kidney genes in *Il18*^+/+^ and *Il18*^−/−^ mice were defined to show signal ratios with a greater than twofold increase or a less than 0.5-fold decrease. Details of microarray analysis and results can be found in Gene Expression Omnibus (Accession No. GSE64310).

IPA software was used for microarray analyses to provide functionality for the interpretation of gene expression data. To investigate molecular mechanism on the kidney by IL-18, core analysis was performed with the setting for “Tissues” being only “Kidney”. This software for molecular analysis was based on that described in previous studies [17, 18].

For RT-qPCR, kidney samples at 6 and 12 weeks of age were obtained from the same mice used for microarray analyses. Total RNA (10 ng/reaction) was used in the RNA-direct SYBR Green Real-Time PCR Master Mix (One-step qPCR Kit; Toyobo Co., Ltd., Tokyo, Japan). Samples were put in duplicate reactions in 384-well plates and run on a QuantStudio 12K Flex PCR system (Thermo Fisher Scientific, Waltham, MA, USA). Median threshold cycle values were used to calculate the fold change between samples from two groups. Fold change values were normalized to glyceraldehyde-3-phosphate dehydrogenase (*Gapdh*). The following temperature profile was used: 30 s at 90 °C and 20 min at 61 °C for reverse transcription, according to the manufacturer’s instructions, followed by 45 cycles at 98 °C for 1 s, 67 °C for 15 s, and 74 °C for 35 s. The primer sequences for RT-qPCR are shown in Additional file 1.

### Short- and long-term treatment of mice with rIL-18

To determine treatment responses to IL-18, mice were administered 2 µg/mouse rIL-18 dissolved in saline containing heat-inactivated normal mouse serum (0.5%). Mice were injected twice a week via the caudal vein for 2 weeks (short-term study) from 10 weeks of age, and for 12 weeks (long-term study) from 37 weeks of age, as previously reported [3]. For control experiments, saline was injected by the same procedure. Five to six and three mice per group were included in the short- and long-term treatment groups, respectively.

### Statistical analysis

All statistical analysis was performed as previously described [3, 8]. Sigmaplot™ (ver. 11.0; Systat Software, Inc., San Jose, CA, USA) was used for statistical analyses. RT-qPCR was analyzed using the Student’s *t* test after the equal variances test or Mann–Whitney *U*-test were performed as appropriate. Equal variances results are expressed as mean ± SD, and Mann–Whitney results as medians and ranges. Serum measurements and effects of rIL-18 administration was analyzed by two-way analysis of variance. A *p*-value < 0.05 was considered statistically significant. All analyses were performed at least in duplicate to confirm the results.

## Results

### Histopathological and serum observations of the kidney

**Fig. 1:**
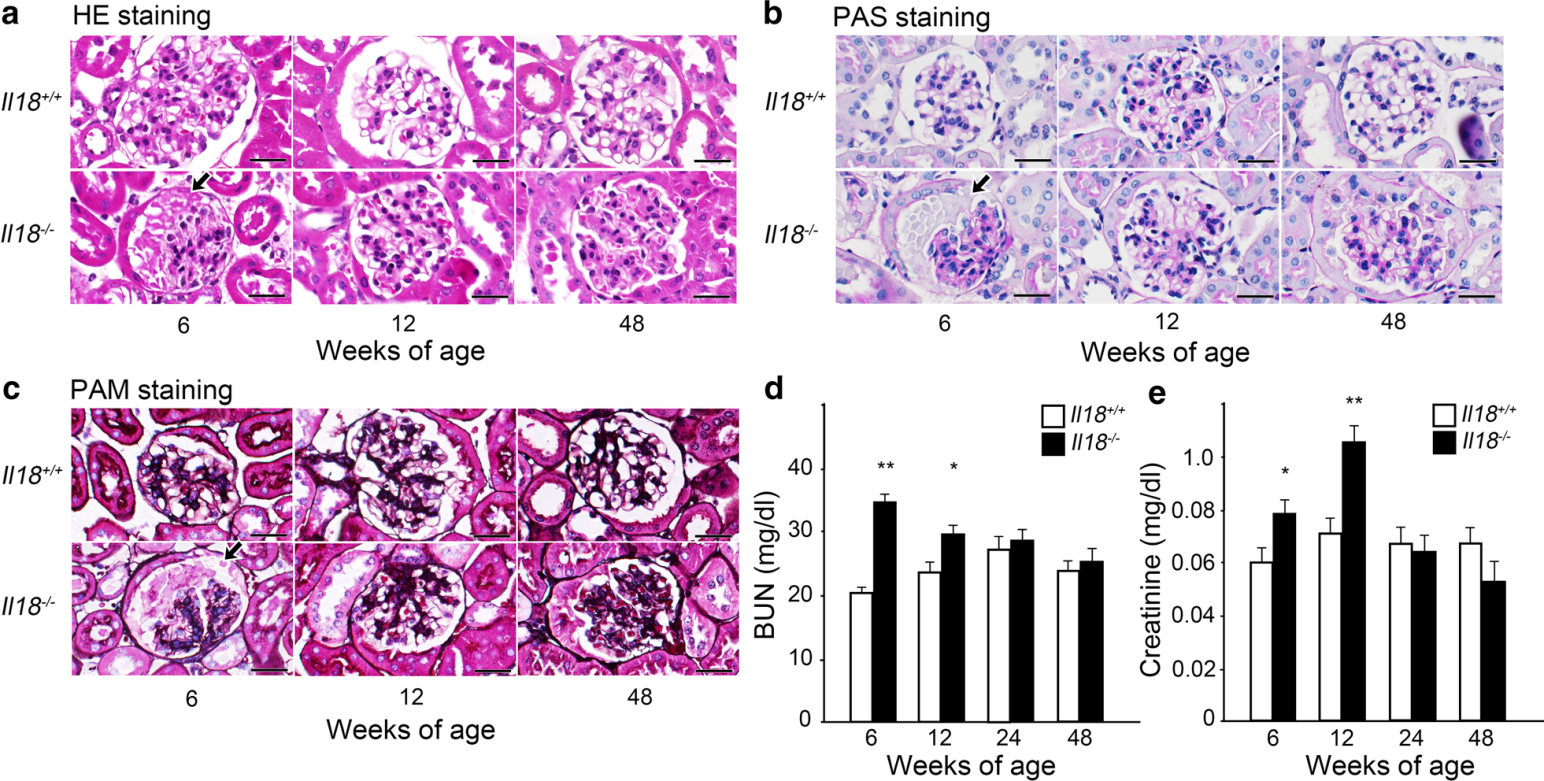
*Il18*^−/−^ mice showed renal impairment in youth but no injury was observed at 48 weeks. **a** Hematoxylin–eosin, **b** periodic acid–Schiff, and **c** periodic acid methenamine silver staining were performed at 6, 12, and 48 weeks of age. Arrows in **a**–**c** at 6 weeks old indicate enucleated epithelial cells of the Bowman’s capsule and the collapse of glomerular capillaries. Scale bars represent 50 μm (**a**–**c**). To assess the degree of damage to the kidney, serum BUN (**d**) and CREA (**e**) were measured (n = 6–8 mice per group). Data are mean ± SD. *p < 0.05, **p < 0.01. BUN: blood urea nitrogen; CREA: creatinine

Histopathological changes were observed in *Il18*^−/−^ mice at 6 weeks of age, especially around glomeruli (Fig. 1a–c, Additional file 2). Enucleated epithelial cells of the Bowman’s capsule and collapse of glomerular capillaries were observed at 6 weeks of age in *Il18*^−/−^ mice, and serum levels of CREA and BUN in *Il18*^−/−^ mice increased compared with *Il18*^+/+^ mice at 6 and 12 weeks of age. As mice aged, there were no comparable differences (Fig. 1d, e). Moreover, in the interstitium there were also no remarkable findings such as macrophage or T cell infiltration, as shown by F4/80 and CD4 staining at 6, 12, and 24 weeks of age (Additional files 2, 3). *Il18*^−/−^ mice at 6 weeks old showed renal impairment but there was no evidence of kidney injury at 48 weeks old between groups.

### Microarray and IPA

To identify the genes responsible for kidney failure (including dyslipidemia and diabetes mellitus but not renal function degeneration) in mice at 6 and 12 weeks old, gene expression profiles in *Il18*^−/−^ mice were compared with control mice at 6 and 12 weeks old. For *Il18*^−*/*−^ mice, 158 genes at 6 weeks and 142 genes at 12 weeks showed a greater than twofold increase or a less than 0.5-fold decrease, respectively (p < 0.05 for both age groups). Among these genes, eight and four molecules at 6 and 12 weeks old, respectively, involved in kidney function were identified from core analysis using IPA. These were paired box gene 2 (*Pax2*), interleukin 18 (*Il18*), integrin alpha M (*Itgam*), peroxisome proliferator activator receptor delta (*Ppard*), lecithin-retinol acyltransferase (*Lrat*), mitogen-activated protein kinase 8 (*Mapk8*), stabilin 2 (*Stab 2*), and nephroblastoma overexpressed gene (*Nov*) at 6 weeks old and *Il18*, C-X-C motif chemokine 10 (*Cxcl10*), Notch homolog 1 (*Notch1*), and cytochrome P450, family 4, subfamily a, polypeptide 14 (*Cyp4a14*) at 12 weeks old (Tables 1, 2, Additional file 1).

**Table 1:** Core analysis of the kidney in 6-week-old mice

Diseases or functions annotation	p-value	Molecules
Delayed hypersensitive reaction of renal glomerulus	4.92E−03	*Il18*
Injury of kidney	3.50E−02	*Il18*, *Ppard*
Injury of endothelial cells	4.92E−03	*Itgam*
Thrombosis of blood	4.92E−03	*Itgam*
Recruitment of neutrophils	9.83E−03	*Itgam*
Accumulation of neutrophils	1.96E−02	*Itgam*
Generation of reactive oxygen species	2.92E−02	*Itgam*
Concentration of retinol	9.83E−03	*Lrat*
Hypertrophy of renal glomerulus	1.27E−02	*Mapk8*, *Stab 2*
Congestion of vasculature	1.96E−02	*Nov*
Aggregation of mesenchymal cells	4.92E−03	*Pax2*
Abnormal morphology of renal calyx	9.83E−03	*Pax2*
Development of renal calyx	9.83E−03	*Pax2*
Organogenesis of kidney	9.83E−03	*Pax2*
Size of renal medulla	9.83E−03	*Pax2*
Hypoplasia of renal cortex	1.47E−02	*Pax2*
Development of epithelial cells	1.96E−02	*Pax2*
Injury of outer medulla	4.92E−03	*Ppard*
Injury of renal cortex	9.83E−03	*Ppard*
Fibrosis of renal glomerulus	3.87E−02	*Stab 2*

**Table 2:** Core analysis of the kidney in 12-week-old mice

Diseases or functions annotation	p-value	Molecules
Mitogenesis of mesangial cells	1.47E−02	*Cxcl10*
Synthesis of DNA	3.87E−02	*Cxcl10*
Migration of mesangial cells	4.35E−02	*Cxcl10*
Renal vascular resistance	1.96E−02	*Cyp4a14*
Flow of blood	4.82E−02	*Cyp4a14*
Delayed hypersensitive reaction of renal glomerulus	4.92E−03	*Il18*
Transformation of kidney cells	1.47E−02	*Notch1*

### Gene expression comparison between groups by RT-qPCR

To confirm mRNA expression by microarray, RT-qPCR was performed. At 6 weeks old, expression of *Itgam*, *Nov*, and *Stab 2* in *Il18*^−/−^ mice significantly increased compared with *Il18*^+/+^ mice (Table 3). Expression of *Il18*, *Lrat*, and *Ppard* significantly decreased in *Il18*^−/−^ mice compared with *Il18*^+/+^ mice (Table 3). At 12 weeks old, expression of *Cxcl10*, *Cyp4a14*, and *Il18* was lower in *Il18*^−/−^ mice compared with *Il18*^+/+^ mice (Table 4).

**Table 3:** Comparison of molecular expression between microarray and RT-qPCR analysis in 6-week-old mice

Gene	Microarray	RT-qPCR
*Il18*	0.280 ± 0.11	0.154 ± 0.024*
*Itgam*	3.02 ± 0.15	1.77 ± 0.39*
*Lrat*	0.478 ± 0.12	0.416 ± 0.068*
*Mapk8*	0.370 ± 0.28	0.991 ± 0.19
*Nov*	2.60 ± 0.31	1.643 ± 0.10*
*Pax2*	2.13 ± 0.28	1.17 ± 0.35
*Ppard*	0.426 ± 0.10	0.572 ± 0.099*
*Stab 2*	2.08 ± 0.21	1.39 ± 0.067*

Comparison between microarray and RT-qPCR analysis at 6 weeks old is shown as a ratio (*Il18*^−/−^/*Il18*^+/+^). Data are mean ± SD. * p < 0.05 (n = 3 per group)

**Table 4:** Comparison of molecular expression between microarray and RT-qPCR analysis in 12-week-old mice

Gene	Microarray	RT-qPCR
*Cxcl10*	0.495 ± 0.50	0.573 ± 0.036*
*Cyp4a14*	0.461 ± 0.10	0.386 ± 0.16*
*Il18*	0.241 ± 0.11	0.129 ± 0.069*
*Notch1*	0.481 ± 0.10	0.740 ± 0.28

Comparison of molecular expression between microarray and RT-qPCR analysis at 12 weeks old is shown as a ratio (*Il18*^−/−^/*Il18*^+/+^). Data are mean ± SD.* p < 0.05 (n = 3 per group)

At 6 weeks old, *Itgam*, *Il18*, *Lrat*, *Nov*, and *Ppard* appeared to be expressed differently with microarray analysis, which might be related to kidney function. At 12 weeks old, *Cxcl10*, *Cyp4a14*, and *Il18* showed similar tendencies in terms of differences between the groups.

To validate the correlation between microarray and RT-qPCR analysis, Spearman’s rank correlation coefficient analysis was performed for each group (6-week-old group: p = 0.0038, rs = 0.881; 12-week-old group: p = 0.0016, rs = 0.827).

### Effect of short-term rIL-18 treatment on kidney function

**Fig. 2:**
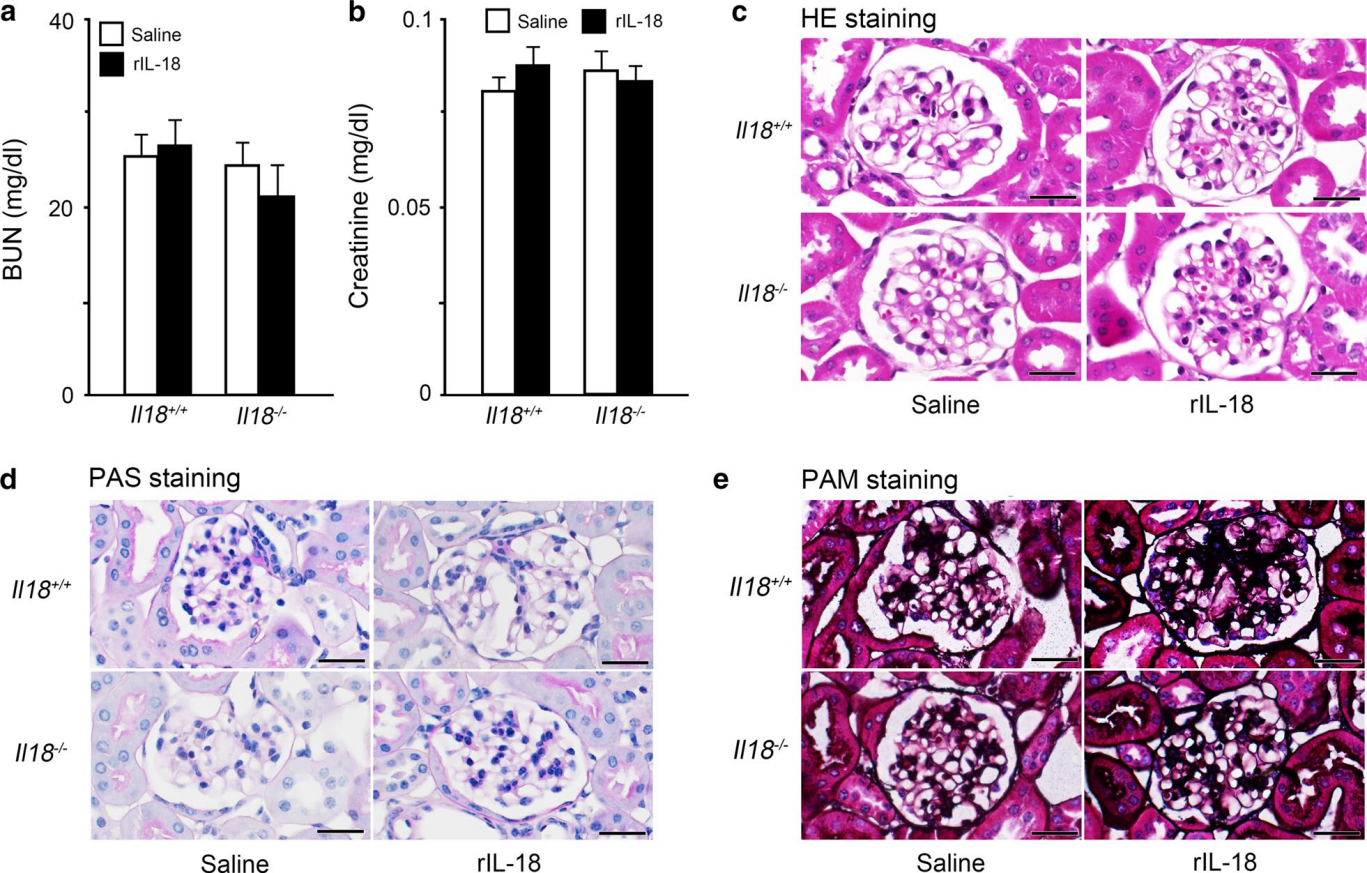
No side effects were observed with rIL-18 administration for 2 weeks. To analyze the effects on renal function, mice were injected with rIL-18 twice a week for 2 weeks from 10 weeks of age (short-term treatment). Serum levels of BUN (**a**) and CREA (**b**) are shown. Data are mean ± SD (**a**, **b**: n = 8 per group). **c** Hematoxylin–eosin, **d** periodic acid–Schiff, and **e** periodic acid methenamine silver staining. Scale bars represent 50 μm (**c**–**e**). BUN: blood urea nitrogen; CREA: creatinine

To verify the effects of IL-18 on the kidney, especially any potential side effects, *Il18*^+/+^ and *Il18*^−/−^ mice were treated with rIL-18. Serum BUN and CREA were not affected by rIL-18 administration (Fig. 2a, b). No histological changes in glomeruli were observed between groups (Fig. 2c–e). Short-term treatment with rIL-18 appeared to have no influence on the kidney, including any side effects.

### Effect of long-term rIL-18 treatment on the kidney in *Il18*^*−/−*^ mice

**Fig. 3:**
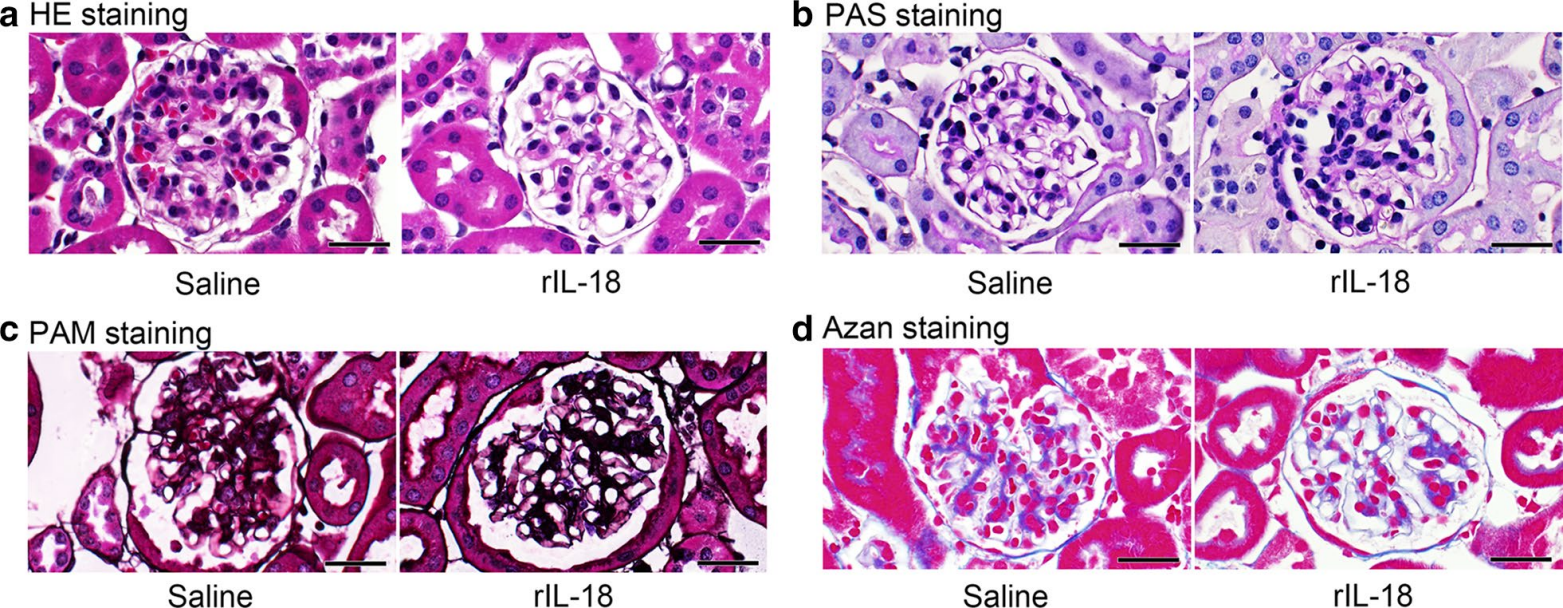
Effects of long-term treatment with rIL-18 on the kidney. Histopathological analysis was conducted to assess renal damage with rIL-18 exposure for 12 weeks. **a** Hematoxylin–eosin, **b** periodic acid–Schiff, **c** periodic acid methenamine silver, and **d** azan staining. Scale bars represent 50 μm (**a**–**d**)

To determine the efficacy of rIL-18 in *Il18*^−/−^ mice, we administered rIL-18 to *Il18*^−/−^ mice twice a week for 12 weeks from 37 weeks old and found that the kidneys of *Il18*^−/−^ mice did not show any differential changes (Fig. 3a–d).

## Discussion

In the current study, we found that: (1) *Il18*^−/−^ mice showed renal impairment in their youth-6 weeks of age, but improved naturally as they aged; (2) even though no renal damage was observed at 48 weeks of age, *Il18*^−/−^ mice showed diabetes mellitus, dyslipidemia, and arteriosclerosis; (3) several molecules related to renal function were affected by a lack of IL-18; and (4) the administration of IL-18 exerted few effects on the kidney regardless of short or long-term administration.

IL-18 is associated with the pathogenesis of a number of renal disorders, such as autoimmune diseases [19, 20]. In humans, IL-18 in the urine is one of the early markers of renal tubular disease [21]. IL-18 deficiency protects against renal fibrosis by aldosterone-salt treatment [22]. In human mesangial cells, inhibition of 5-lipoxygenase and cyclooxygenase, which play important roles in the pathogenesis of glomerulonephritis in childhood, resulted in IL-18-induced proinflammatory cytokine release and cellular proliferation of these cells [23–32]. It is possible that IL-18 has a substantial impact on the kidney, including renal tubules, glomeruli, and mesangial cells.

No remarkable changes were observed in renal tubules during initial growth in the current study. In youth, however, enucleated epithelial cells of the Bowman’s capsule and collapse of glomerular capillaries were detected despite a deficiency of IL-18 (Fig. 1a–c). Serum BUN levels in *Il18*^−/−^ mice increased significantly compared with *Il18*^+/+^ mice. These results suggest that a deficiency in IL-18 led to temporary kidney damage during youth, however this damage might improve naturally over time.

*Il18*^−/−^ mice show dyslipidemia at 6 weeks old, develop diabetes mellitus, and show high glucose and insulin levels and arteriosclerosis at 6 months old, and NAFLD or NASH at 48 weeks old [3, 8]. Structural and functional changes in the kidney develop with diabetes mellitus [12]. In the current study, at over 6 months old, no remarkable changes to the kidney were observed (Fig. 1a–e). Down-regulation of IL-18 expression can protect renal function and prevent the development of diabetic nephropathy [33]. It is suggested that IL-18 deficiency can result in an energy unbalance, such as some sort of metabolic disorder, despite preserving renal function.

With regard to possible molecular mechanisms, eight and four genes at 6 and 12 weeks old, respectively, were identified by core analysis using IPA (Tables 1, 2). Expression of *Il18*, *Itgam*, *Lrat*, *Nov*, *Ppard*, and *Stab 2* were significantly different between *Il18*^+/+^ and *Il18*^−/−^ mice at 6 weeks old using RT-qPCR (Table 3). Similar to that observed in 6-week-old mice, 12-week-old mice showed significantly different expression of *Cxcl10*, *Cyp4a14*, and *Il18* between groups (Table 4).

At 6 weeks of age, renal impairment was observed in *Il18*^−/−^ mice. As reported previously, inhibition of IL-18 protects renal function, such as prevention of the development of diabetic nephropathy [33]. *Itgam* induces kidney macrophage recruitment, and glomerular histological changes, and contributes to kidney injury in diabetic nephropathy [34]. *Nov* shows reduced expression levels with inflammation and renal fibrosis after nephropathy in mice [35]. *Ppard* plays an important role in energy metabolism and *Ppard* agonist decreases insulin and glucose levels by increasing glucose transport and possibly affecting subsequent chronic kidney disease risks [36]. Polymorphisms in *PPARD* is significantly associated with the risk for chronic kidney disease in Japanese [36]. A deficit in *Stab 1* and *Stab 2* exhibit the development of severe glomerular fibrosis [37]. In the current study, expression of *Itgam*, *Nov*, and *Stab 2* increased and *Ppard* decreased in *Il18*^−/−^ mice (Table 3), suggesting that increased levels of *Itgam* and *Nov* and decreased expression of *Ppard* led to kidney damage in youth-aged mice, although high levels of *Stab 2* might show some protective effects.

At 12 weeks old, renal impairment showed signs of improvement compared with 6-week-old mice (Fig. 1a–e). The affected molecules at 6 weeks old normalized, excluding *Il18*, and expression of *Cxcl10* and *Cyp4a14* in *Il18*^−/−^ mice was lower compared with *Il18*^+/+^ mice (Table 4). CXCL10/*Cxcl10* can be expressed by mesangial cells [38, 39] and tubular epithelial cells [40] in the kidney by stimulation of proinflammatory cytokines, such as interferon-γ [38]. *Cyp4a14*-deficient mice exhibit deterioration of renal disease with increased albuminuria, mesangial expansion, and glomerular matrix deposition [41]. Consequently, loss of IL-18 might result in loss of the ability to induce normal inflammatory responses or to decrease *Cxcl10* levels or to protect the kidney.

In a previous study, we found that dyslipidemia in *Il18*^−/−^ mice recovered with short-term (2 weeks) administration of rIL-18 [3]. Additionally, *Il18*^−/−^ mice that received rIL-18 for 12 weeks recovered from conditions corresponding to NAFLD or NASH [3]. In another study, we suggested a new cancer immunotherapy using IL-18 [14]. Therefore, it appears that IL-18 is not only essential for the synthesis of lipids, for maintaining an energy balance, and for promoting normal lipolysis, but also plays a role in boosting immunological functions.

Before clinical application, the effects of IL-18 on the kidney require study. We found that short-term intravascular administration of IL-18 (2 weeks) induced no kidney damage, and appeared to improve dyslipidemia in *Il18*^−/−^ mice (Fig. 2a–e) [3]. With long-term administration of rIL-18 in *Il18*^−/−^ mice, we observed no remarkable findings (Fig. 3a–d). These results suggest that IL-18 administration had little effect on the kidney in these mice, but did show a number of beneficial effects, such as maintaining energy balance and cancer immunotherapy.

This study was limited in that only IL-18 and no other medication was administered. It is possible that IL-18 combined with other medication may have other effects on the kidney including side effects. Moreover, this study only focused on the kidney. However, we are currently investigating the relationship between IL-18 and physiological homeostasis [3], with particular emphasis on not only other remodeling of the kidney but also other organs such as adipose tissue and the pancreas.

## Conclusion

We observed a novel influence of IL-18 on the kidney. A deficiency in IL-18 resulted in kidney injury in younger mice but protection from renal damage was observed in mice aged 48 weeks even though *Il18*^−/−^ mice showed signs of diabetes mellitus, dyslipidemia, and arteriosclerosis. Because IL-18 is considered a possible novel clinical treatment for some conditions, such as cancer immunotherapy, assessment of adverse effects are required. In this study, we found that short- and long-term administration of IL-18 had no adverse effects on the kidney in this population of mice.

## Additional files

**Additional file 1.** Primer sequences used for RT-qPCR. Primer sequences for each gene are shown.

**Additional file 2.** HE staining of the interstitium and azan staining of the kidney at 6, 12, and 48 weeks of age. (a) HE staining of the renal interstitium. (b) Histopathological azan staining of the kidney. Scale bars represent 50 μm. HE: Hematoxylin and eosin.

**Additional file 3.** No macrophages were observed in the kidney at 6, 12, and 48 weeks of age. Immunostaining findings of (a) F4/80 and (b) CD4 are shown during aging. Scale bars represent 50 μm.

## Abbreviations

BUN: blood urea nitrogen
CREA: creatinine
*Cxcl10/*CXCL10: C-X-C motif chemokine 10
*Cyp4a14*: cytochrome P450, family 4, subfamily a, polypeptide 14
*Gapdh*: glyceraldehyde-3-phosphate dehydrogenase
IL-18: interleukin 18
*Il18*: interleukin 18
*Il18*^−/−^: IL-18 knockout
IPA: Ingenuity^®^ Pathway Analysis
*Itgam*: integrin alpha M
*Lrat*: lecithin-retinol acyltransferase
*Mapk8*: mitogen-activated protein kinase 8
NAFLD: non-alcoholic fatty liver disease
NASH: non-alcoholic steatohepatitis
*Notch1*: Notch homolog 1
*Nov*: nephroblastoma overexpressed gene
*Pax2*: paired box gene 2
*Ppard*/*PPARD*: peroxisome proliferator activator receptor delta
RT-qPCR: quantitative reverse transcription polymerase chain reaction
rIL-18: recombinant IL-18
*Stab 2*: stabilin 2
