# Supplementary material for: Physiological and molecular effects of interleukin-18 administration on the mouse kidney
Source: J Transl Med. 2018 Mar 7;16:51. doi: 10.1186/s12967-018-1426-6 (PMC5842592; doi:10.1186/s12967-018-1426-6)
Supplement: Supplementary file 1 — Additional file 1. Primer sequences used for RT-qPCR. Primer sequences for each gene are shown. [file 12967_2018_1426_MOESM1_ESM.docx]

**Additional file 1. Primer sequences used for qRT-PCR.**

| **Gene** | **GenBank Accession** |  | **Primers ( 5' - 3' )** |
| --- | --- | --- | --- |
| *Cxcl10* | AK156907 | sense | TAAACGTCCCTCCCGTAACCAC |
|  |  | anti-sense | TAGAGTCACAGACCCGTCCC |
| *Cyp4a14* | NM_007822 | sense | TGGACATCCTCTTGTTTGCCAG |
|  |  | anti-sense | ACGCAGGTCCTCATCAGACA |
| *Gapdh* | NM_008061 | sense | TCTTAAAGAGACTGTGGGCATCAATCTCC |
|  |  | anti-sense | GCGTTGTCCAAACAGAATCCACT |
| *Il18* | NM_008360 | sense | AGGACAAAGAAAGCCGCCTC |
|  |  | anti-sense | TCATTTCCTTGAAGTTGACGCAAGAGT |
| *Itgam* | NM_008401 | sense | GCCAGAACCCGCTCACCAA |
|  |  | anti-sense | TTCAGAGCCCCATGCCCTT |
| *Lrat* | NM_023624 | sense | AGAACCGTCCCTATGAAATCAGC |
|  |  | anti-sense | AAAATGGGTCCGTGACACCT |
| *Mapk8* | NM_016700 | sense | TAGCCCTTACTGTTTGACCT |
|  |  | anti-sense | GGTTTGCACTTCACATGCTT |
| *Notch1* | NM_008714 | sense | CTCAAGCCCCTGAAGAATGCC |
|  |  | anti-sense | CTTCGTCTCCCCACTCGTTC |
| *Nov* | NM_010930 | sense | CTGACTCCAGCATTAACTGCAT |
|  |  | anti-sense | TGGACACGCCCATTCCACA |
| *Pax2* | NM_011037 | sense | CCTGCGAGCCGACACC |
|  |  | anti-sense | AGGACGCTCAAAGACTCGAT |
| *Ppard* | NM_011145 | sense | ATCCTCACCGGCAAGTCCA |
|  |  | anti-sense | CCTGCCACAGTGTCTCGATG |
| *Stab2* | AK142339 | sense | CTCTGCCATCCTCTCACATGC |
|  |  | anti-sense | TGATATTCCCATCTGCCTTGTCC |
