# Supplementary figures and images for: Physiological and molecular effects of interleukin-18 administration on the mouse kidney
Source: J Transl Med. 2018 Mar 7;16:51. doi: 10.1186/s12967-018-1426-6 (PMC5842592; doi:10.1186/s12967-018-1426-6)

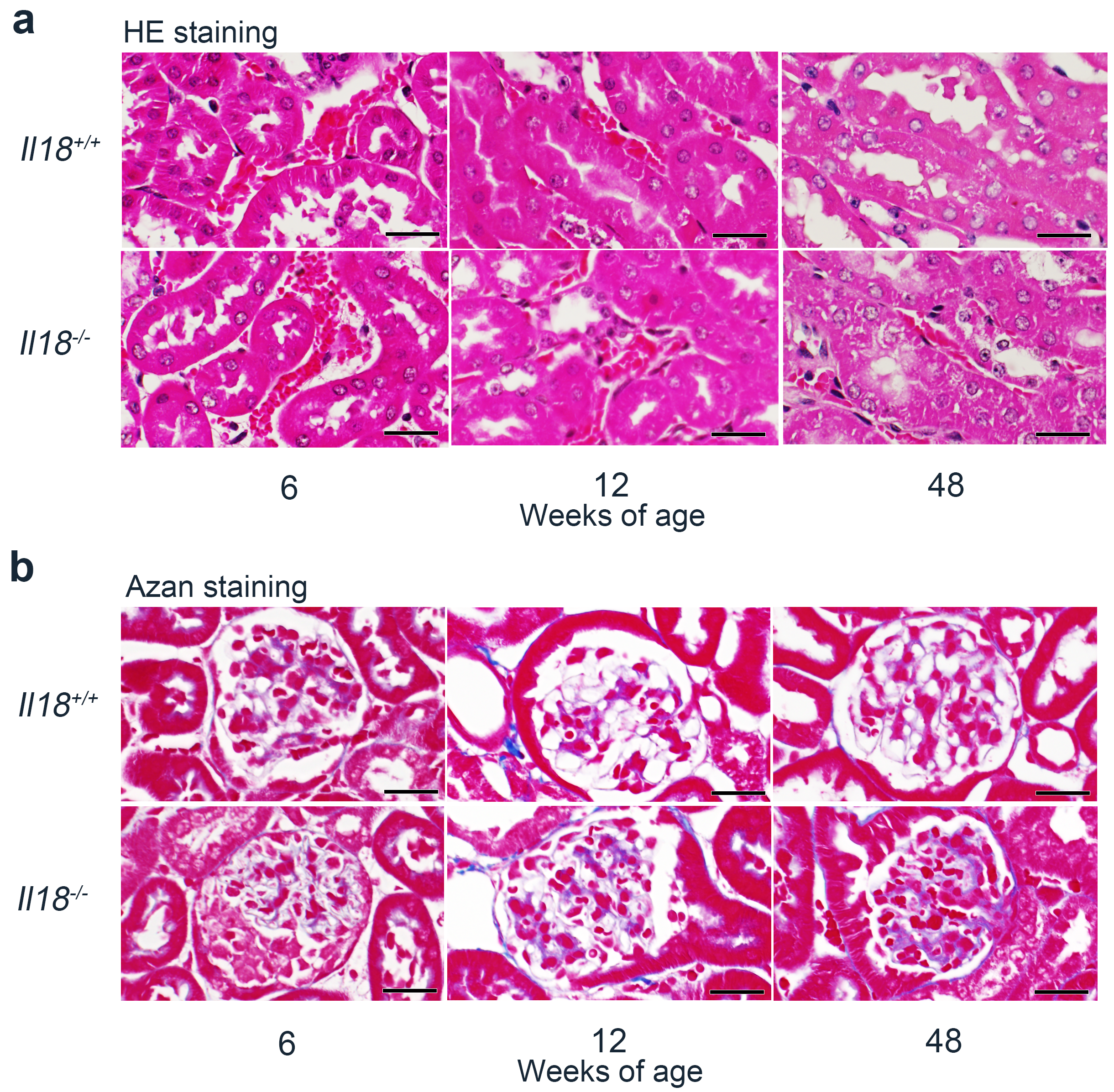

Supplement: Supplementary file 2 — Additional file 2. HE staining of the interstitium and azan staining of the kidney at 6, 12, and 48 weeks of age. (a) HE staining of the renal interstitium. (b) Histopathological azan staining of the kidney. Scale bars represent 50 μm. HE: Hematoxylin and eosin. [file 12967_2018_1426_MOESM2_ESM.tif]

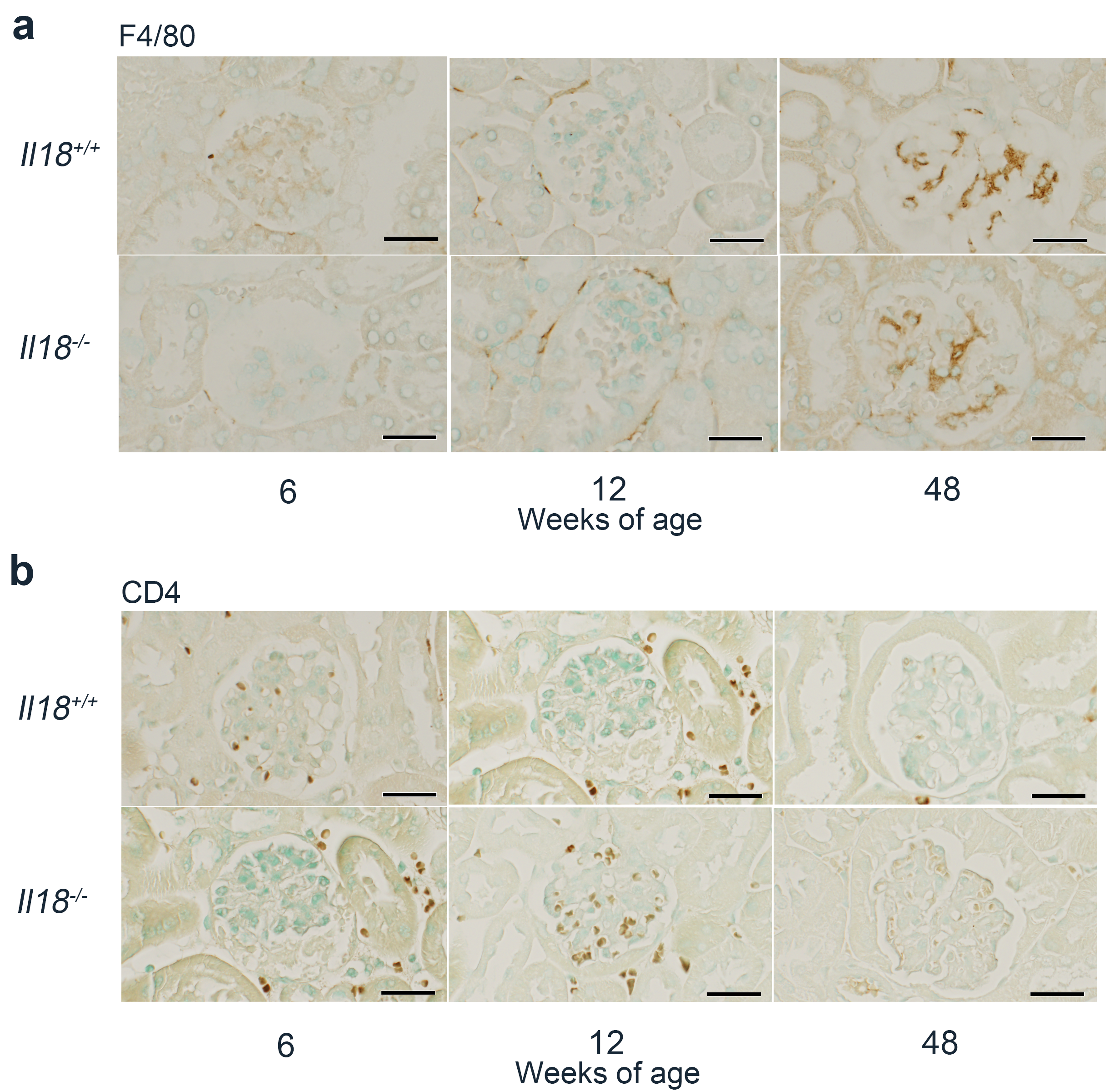

Supplement: Supplementary file 3 — Additional file 3. No macrophages were observed in the kidney at 6, 12, and 48 weeks of age. Immunostaining findings of (a) F4/80 and (b) CD4 are shown during aging. Scale bars represent 50 μm. [file 12967_2018_1426_MOESM3_ESM.tif]
